# Supplementary figures and images for: Targeting CCRL2 enhances therapeutic outcomes in a tuberculosis mouse model
Source: Front Immunol. 2025 Mar 20;16:1501329. doi: 10.3389/fimmu.2025.1501329 (PMC11965133; doi:10.3389/fimmu.2025.1501329)

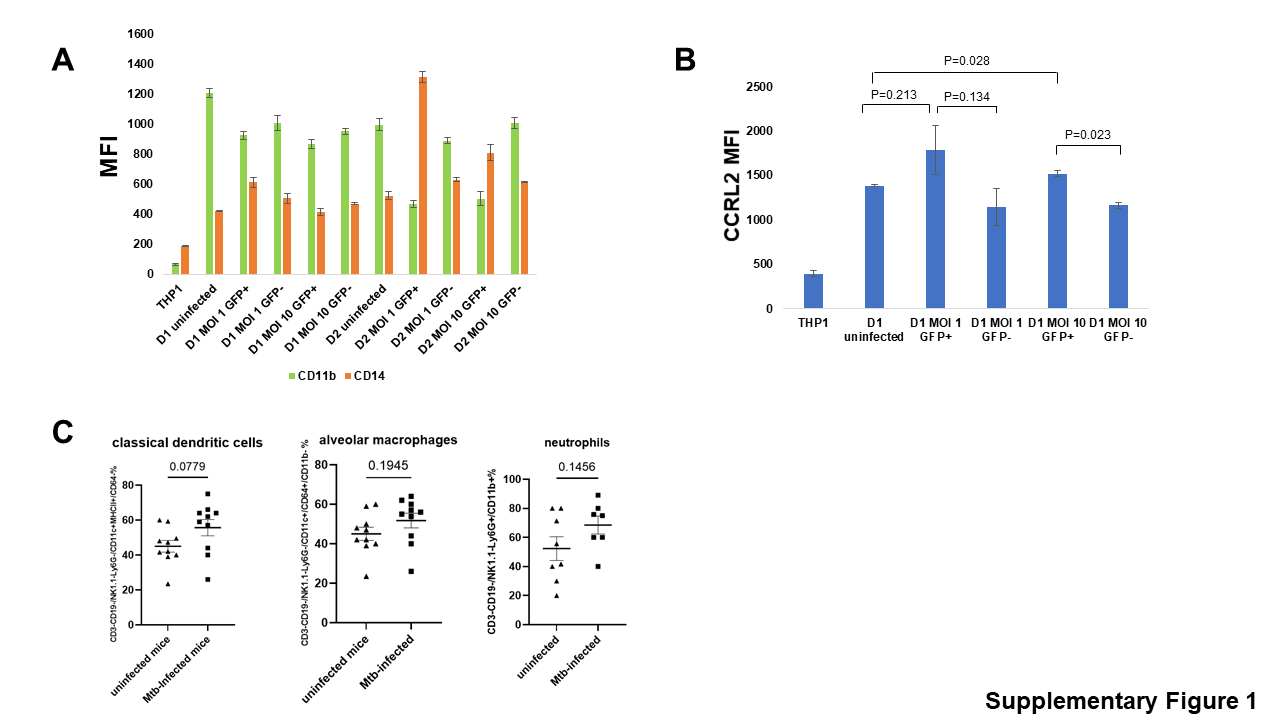

Supplement: Supplementary Figure 1 — (A) CD11b+ and CD14+ expression (measured as MFI through flow cytometry) were used to confirm differentiation of THP-1 into macrophages treated with PMA (B) THP-1 cells were infected with the GFP+ Mtb strain at MOI of 1 and 10 following treatment with PMA to induce differentiation to macrophages and were incubated for 1 day. Using flow cytometry, CCRL2 expression (MFI) was measured across the different conditions. These experiments were repeated three times, and one-way analysis of variance followed by Tukey’s multiple comparisons tests was used; (C) populations (%) of cDCs, alveolar macrophages, and neutrophils derived from mouse lungs collected from Mtb-infected vs. uninfected mice; Flow cytometry experiments was repeated at least twice and unpaired T-test was used; MOI, Multiplicity of infection; GFP, green fluorescent protein; D2, Day 2; MFI, Median fluorescent intensity; Mtb, Mycobacterium tuberculosis, CFU, colony-forming units; Classical DCs, classical dendritic cells. [file Image1.tif]

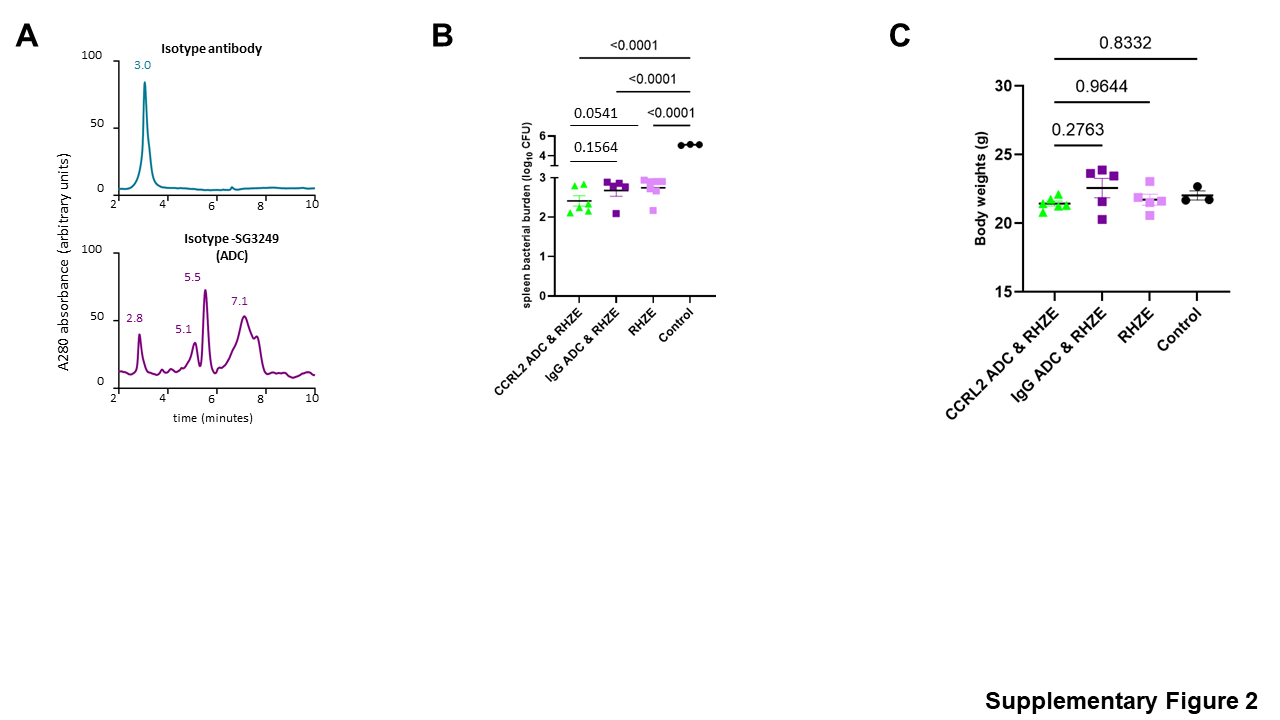

Supplement: Supplementary Figure 2 — (A) Hydrophobic interaction chromatography (HPLC) of isotype control antibody and isotype control-SG3249 ADC; (B) Scatterplot of spleen mycobacterial burden in mice at 7 weeks after RHZE (n=20); (C) Mouse body weights (g) at 7 weeks after RHZE initiation (n=20). One-way analysis of variance followed by Tukey’s multiple comparisons tests was used; Mtb, Mycobacterium tuberculosis, RHZE, Rifampin-Isoniazid-Pyrazinamide-Ethambutol; ADC, Antibody-drug conjugate; CFU, colony-forming units. [file Image2.tif]

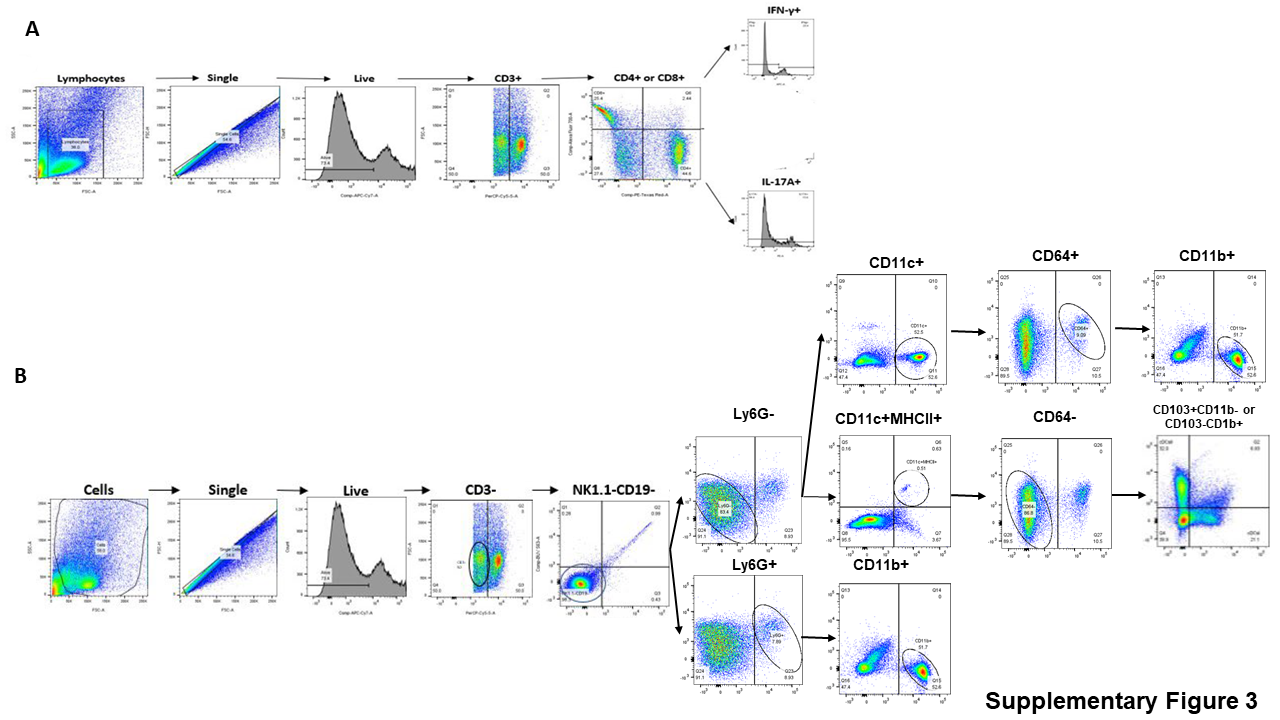

Supplement: Supplementary Figure 3 — Two-dimensional gating strategy for flow cytometric identification of (A) T-cell-producing cytokines, (B) DCs, alveolar macrophages, and neutrophils. We excluded doublets and debris and gated on single live cells. (A) We identified T cells (CD3+), CD4+ T cells, CD8+ T cells, and T-cell producing cytokines (CD3+CD4+ IFN-γ+, or IL-17A+ and CD3+CD8+ IFN-γ+, or IL-17A+); (B) classical DCs (CD3-CD19-NK1.1-Ly6G-MHCII+CD11c+) and non-lymphoid tissue cDCs I (CD3-CD19-NK1.1-Ly6G-MHCII+CD11c+CD64-CD103+CD11b-) and cDCs II (CD3-CD19-NK1.1-Ly6G-MHCII+CD11c+CD64-CD103-CD11b+); alveolar macrophages (CD3-CD19-NK1.1-Ly6G-CD11c+CD64+CD11b+); and neutrophils (CD3-CD19-NK1.1-Ly6G+CD11b+); DCs, total classical dendritic cells; cDCs I, classical dendritic cells type I; cDCs II, classical dendritic cells type II. [file Image3.tif]
